# Safer injections following a new national medicine policy in the public sector, Burkina Faso 1995 – 2000

**DOI:** 10.1186/1471-2458-5-136

**Published:** 2005-12-19

**Authors:** Sophie Logez, Yvan Hutin, Paul Somda, Jérôme Thuault, Kathleen Holloway

**Affiliations:** 1 Department of Medicines Policy and Standards, World Health Organization Headquarters, Geneva, Switzerland; 2 World Health Organization, Resident Adviser, Chennai, India; 3 Department of General Inspection for Health Care Facilities, Ministry of Health, Ouagadougou, Burkina Faso; 4 Department of Public Health, Agadez, Niger; 5 Department of Medicines Policy and Standards, World Health Organization Headquarters, Geneva, Switzerland

## Abstract

**Background:**

The common failure of health systems to ensure adequate and sufficient supplies of injection devices may have a negative impact on injection safety. We conducted an assessment in April 2001 to determine to which extent an increase in safe injection practices between 1995 and 2000 was related to the increased access to injection devices because of a new essential medicine policy in Burkina Faso.

**Methods:**

We reviewed outcomes of the new medicine policy implemented in1995. In April 2001, a retrospective programme review assessed the situation between 1995 and 2000. We visited 52 health care facilities where injections had been observed during a 2000 injection safety assessment and their adjacent operational public pharmaceutical depots. Data collection included structured observations of available injection devices and an estimation of the proportion of prescriptions including at least one injection. We interviewed wholesaler managers at national and regional levels on supply of injection devices to public health facilities.

**Results:**

Fifty of 52 (96%) health care facilities were equipped with a pharmaceutical depot selling syringes and needles, 37 (74%) of which had been established between 1995 and 2000. Of 50 pharmaceutical depots, 96% had single-use 5 ml syringes available. At all facilities, patients were buying syringes and needles out of the depot for their injections prescribed at the dispensary. While injection devices were available in greater quantities, the proportion of prescriptions including at least one injection remained stable between 1995 (26.5 %) and 2000 (23.8 %).

**Conclusion:**

The implementation of pharmaceutical depots next to public health care facilities increased geographical access to essential medicines and basic supplies, among which syringes and needles, contributing substantially to safer injection practices in the absence of increased use of therapeutic injections.

## Background

Injections are one of the most common medical procedures, with an estimated 16 thousand million injections administered each year in developing and transitional countries, most of which are for therapeutic purposes [1]. In these developing and transitional countries, WHO estimates that 39% of injections are administered with syringes and/or needles re-used in the absence of sterilization [1]. WHO's Global Burden of Disease study suggests that annually, in developing and transitional countries, 32% of new hepatitis B virus (HBV) infections, 40% of new hepatitis C virus (HCV) infections and 5% of new human immunodeficiency virus (HIV) infections are attributable to contaminated health-care injections [2]. To prevent injection-associated infections, WHO recommends a policy based upon (1) behaviour change among patients and health care workers to reduce injection overuse and improve safety, (2) provision of injection devices and infection control supplies and (3) sharps waste management.

The common failure of health systems to ensure adequate and sufficient supplies of injection devices may have a negative impact on injection safety. The WHO model list of essential medicines made no mention of the need to supply injection devices in quantities that matched supplies of essential injectable medicines although 44% of active ingredients were mentioned in injectable form [3]. In a system analysis conducted in 1995, the logistics project of WHO's African Regional Office (AFRO) identified the failure to systematically fund sufficient supplies of injection devices as part of immunization services as a key determinant of widespread reuse of syringes and needles in absence of sterilization [4]. In 2000, WHO, the United Nations Children's fund (UNICEF), the United Nations Population Fund (UNFPA) recommended in a Joint Statement that sufficient syringes and safety boxes be supplied with consignments of vaccines to address the issue of insufficient supplies of injection devices for immunization purposes [5]. In the curative health care sector where 95% of all injections are provided, the concept of injection device security entails that injectable medicines, diluents, single-use injection devices and safety boxes are supplied in timely manner and adequate quantities [6]. However, it has not yet been implemented on the large scale.

**Figure 1:**
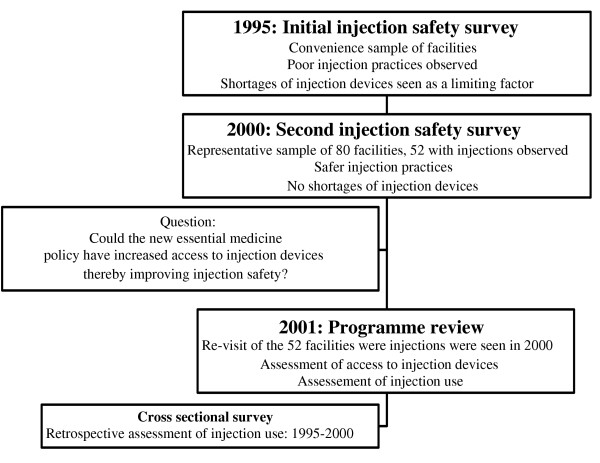
Sequence of the injection safety assessments in Burkina Faso that led to the programme review reported in this article, 1995–2001.

Burkina Faso is a good setting to examine how access to injection devices impacts on injection safety. In 1995, an injection safety assessment indicated that (1) sterile injection devices were used for each injection in 80% of the urban health care facilities, 60% of provincial facilities and 11% of the rural facilities and (2) up to 48% of health care facilities visited reported insufficient quantities of injection devices available [4]. In contrast, the results of a second assessment carried out in 2000 on a sample representative of the facilities in the country suggested that practices had substantially improved [7]. In 96% of the 52 health care facilities visited, a new syringe and a new needle were used for each patient and that there were no shortages of injection devices. At 49 of the 52 health care facilities (94%), injection devices needed to administer injectable medicines were systematically prescribed for treatment. A discussion of the results generated the hypothesis that the new national essential medicine policy that promoted a better access to medicines and single-use injection devices had made an important and substantial contribution to safer injection practices [8-10]. In April 2001, we conducted a programme review to better document how the new national essential medicines policy had contributed to safer injection practices (Figure 1). The objectives of this programme review were (1) to identify the features of the national essential medicines policy that might have influenced injection practices, (2) to quantify the improved access to injection devices between 1995 and 2000, (3) to determine whether an increased access to injection devices could have led to irrational use of injections and (4) to identify potential adverse effects of the increased availability of injection devices on sharps waste management.

## Methods

### Survey design and sampling

We conducted a cross-sectional survey in 2001 to assess retrospectively the situation between 1995 (the year of the initial injection safety survey that was also the year of implementation of the new national policy to improve access to essential medicines) and 2000 (the year of the survey suggesting better injection practices, Figure 1) [11,12]. For this 2001 programme review, we sampled and re-visited the 52 health care facilities where injections had been observed among the 80 included in the 2000 injection safety assessment. The 2000 injection safety assessment used a two-stage cluster sample made of eight clusters of 10 health care facilities. At each of the 52 facilities visited in April 2001, we selected randomly 30 prescriptions for each month of June between 1995 and 2000 and visited the adjacent public pharmaceutical depots. Finally, we interviewed the managers of the 11 district wholesalers supplying the 52 facilities included in the sample.

### Data collection

Standardized data collection included (1) structured observations of injection devices available in the health facility, (2) reviews of the registers to estimate the proportion of prescriptions including at least one injection during the months of June between 1995 and 2000, (3) interviews of health care workers using standardized questionnaires, (4) interviews of operational public pharmaceutical depots managers using standardized questionnaires, (5) structured observations of 5 ml syringes with needle and 15 basic essential medicines available in the depot (chosen as sentinel indicators of availability and good stock management) and (6) structured interviews of district wholesalers managers. We recorded the origin and brand name of injection devices observed and the sale prices of syringes and needles in the pharmaceutical depots. Seven teams of two investigators collected the information. All teams standardized their data collection procedure before the fieldwork through training and a field visit.

### Other information regarding injection devices

We interviewed the manager of the national wholesaler who provided figures estimating the number of injection devices sold annually in the public health care sector and fixed retail prices. We converted the retail prices of syringes and needles in the public sector set by the Ministry of Health in US$ according to the exchange rate in use in May 2001 (1 US$ for 750 Francs CFA). We used the standard set of medicines and basic consumables given to open a pharmaceutical depot in Burkina Faso by Nongovernmental Organizations (NGOs) with the Ministry of Health approval to estimate the proportion of the essential medicines expenditure used to procure injection devices. This standard package was representative of a typical consumption of a depot and covered the need of a population of 10 000 persons for three to six months in Burkina Faso. Finally, we reviewed the major changes in the new national essential medicines policy, particularly related to access to medicines and basic supplies, including the number of operational pharmaceutical depots set up throughout the country annually and the regulation of medicine retailed prices in the public sector.

### Data analysis

We entered and analysed data using the version 1.2d of the Sphinx plus software (Le Sphinx Développement, Chavanod, France). Proportions were calculated using the number of health care facilities visited (52), the number of pharmaceutical depots or the number of health care workers interviewed as denominator, as appropriate.

### Protection of human subjects

The Ministry of Health approved the protocol and provided an introduction letter for the visit of health care facilities. We met each regional director prior to field visits in each area. However, there was no communication with the health care facilities prior to the arrival of the field workers. We informed participants about the purpose of the assessment and about their right to refuse. Participation was voluntary for all interviewed. When injections were about to occur with non-sterile devices, field workers were asked to interrupt tactfully the procedure. All information was collected confidentially using codes.

## Results

### Improved geographical access to community pharmacies and injection devices

**Figure 2:**
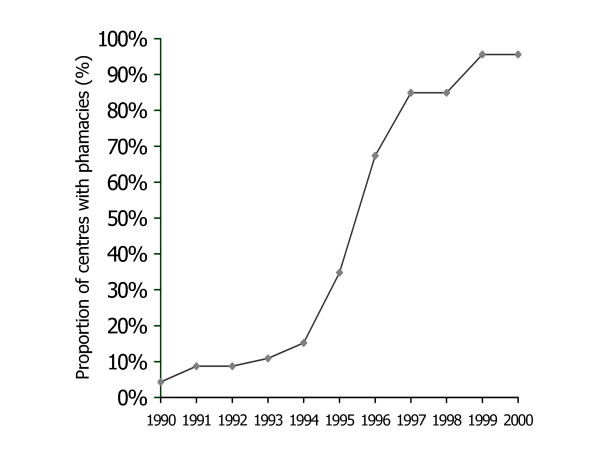
Proportion of 52 health care facilities visited in 2001 that were equipped with a functional pharmaceutical depot, 1990 – 2000, Burkina Faso. (Assessed through asking the date of opening of the pharmaceutical depot in each of the health care facilities visited).

Geographical access to injection devices improved substantially between 1995 and 2000. Of the 52 health care facilities visited in April 2001, 50 (96%) were equipped with a public sector pharmaceutical depot selling syringes and needles directly to patients. Of these, 37 (74%) had been established between 1995 and 2000 (Figure 2). For the two remaining facilities, one had a private depot supplied by private distribution mechanisms and the other had a non-operational depot. Of 50 public pharmaceutical depots visited, 48 (96%) had single-use 5 ml syringes available. An average of 12 out of the 15 key essential medicines selected from the national drug formulary (79%) were actually in stock in the 50 depots at the time of the assessment. At all health care facilities, patients were buying syringes and needles at the depot for the injections that had been prescribed at the dispensary. Overall, the number of single-use 5 ml syringes sold and distributed by the national wholesaler to the network of public pharmaceutical depots increased from 880,000 in 1996 to 1,840,000 in 2000.

### Price of injection devices and user fees

**Figure 3:**
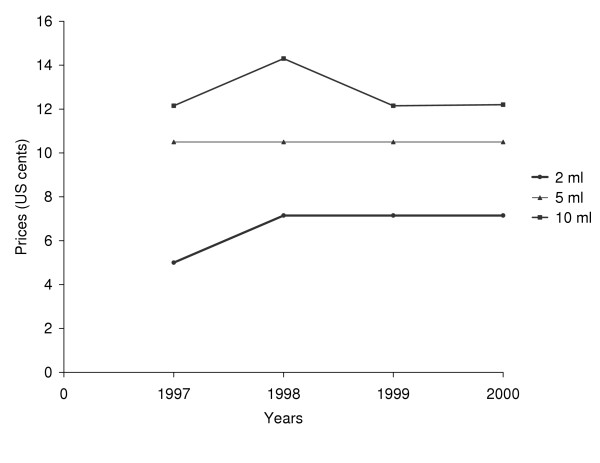
Official retail price of 2 ml, 5 ml and 10 ml syringes and needles set, 1997 – 2000, at pharmaceutical depots, Burkina Faso (obtained from the national wholesaler).

The Ministry of Health fixed the prices of injection devices nationally. The retail price of 2 ml, 5 ml and 10 ml syringe and needle sets did not vary between 1997, 1998 and 2000 (About 10 US$ cents for 5 ml syringes, Figure 3). Of the 52 health care facilities visited, six centres (12%) charged a flat fee for the procedure of injection administration that ranged from three to 26 US$ cents. In 2000, the cost of single-use syringes and needles represented 32 US$ (2.2%) of the 1,430 US$ total cost of a start-up set of medicines and medical supplies given to primary health care facilities to open a pharmaceutical depot.

### Rational use of medicines and sharps waste management

**Figure 4:**
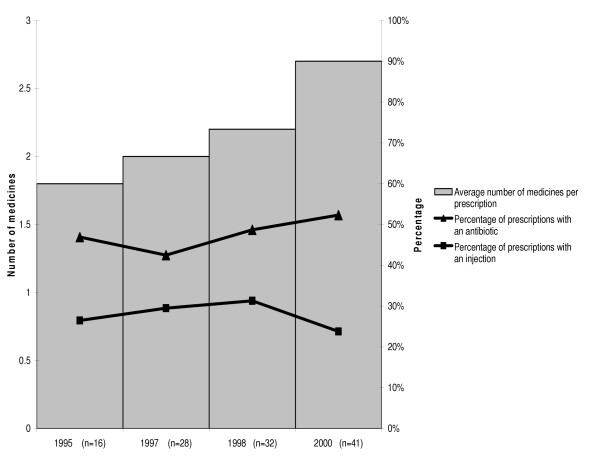
Rational drug use indicators during the months of June in 52 primary health care facilities of Burkina Faso visited in 2001, 1995–2000 (30 prescriptions reviewed in each facility, n = number of health care facilities for which data was available, year by year).

While injection devices were available in greater quantities, the proportion of prescriptions including at least one injection remained stable around 25% between 1995 and 2000 (Figure 4). In contrast, the proportion of prescriptions including at least one antibiotic increased between 1995 and 2000 and the average number of medicines per prescription increased from 1.8 in 1995 to 2.7 in 2000. Of the 52 health care workers interviewed (one per health facility), 37 (71%) participated in an in-service training course on rational use of medicines and 12 (23%) attended a training course that also addressed good injection practice. With respect to sharps waste management, we observed used sharps in the vicinity of 46 (88%) of the 52 health care facilities visited.

## Discussion

Despite differences in methodologies, the results of the consecutive injection safety assessments suggested an improvement of injection practices between 1995 and 2000 in Burkina Faso. The implementation of pharmaceutical depots next to health care facilities increased geographical access to essential medicines and syringes and needles and contributed to an increased use of single-use injection devices. Since the retail prices of injection devices remained unchanged, the improvement in the use is likely to be a consequence of an improved geographical access rather than a consequence of improved financial access. Use of therapeutic injections did not increased substantially during the same period as a result of the increased availability of injection devices. However, use of other medicines, including antibiotics, did increase as the availability of medicines increased in the depots. The different trends observed for antibiotics and injections would suggest that increased availability of injection devices did not lead to overuse of injections.

The number of pharmaceutical depots established in health care facilities dramatically increased between 1995 and 2000. In 1995, the Ministry of Health of Burkina Faso reformed the national essential medicines policy to establish pharmaceutical depots for the delivery of essential medicines and essential medical consumables including injection devices. In 1998, the Ministry of Health further supported this move and decided to systematically set up pharmaceutical depots for all new health care facilities. The strong commitment of the government to improve geographical and financial access to essential medicines and consumables increased access to injection devices. These pharmaceutical depots were managed according to the Bamako Initiative's cost recovery scheme. In Burkina Faso, the distribution system supplies injection devices and injectable medicines through the same channel. This allows supplying matching quantities of injection devices and injectable medicines in the same way that immunization services supply vaccines, auto-disable syringes and safety boxes together as per the "bundling" principles [5].

To maintain prices affordable for the majority of the community, the Ministry of Health fixed retail prices of essential medicines and consumables included in the national list of essential medicines and reviewed those on an annual basis. As a result, the retail price of single-use syringes and needles sets remained stable from 1997 to 2001. In addition, to improve affordability for the population, the government decided the exoneration of importation taxes for essential medicines and essential consumables included in the national list of essential medicines. As retail prices remained stable since 1997, the improvement of the geographical access to injection devices is probably the key factor that contributed to the increased use of single-use injection devices to administer injections in primary health care.

The investment made to procure single-use syringes and needles together with essential medicines was not of a high magnitude. Our analysis suggests that it represented a small proportion (2.2%) of the essential medicines expenditure [3]. In the curative sector, the incremental cost of a set of syringes and needles to ensure injection safety (5 cents US$ per set as international retail price) is moderate (around 10%) when compared with the average cost of an injectable medicine (the median international retail price for 104 injectable medicines included in the WHO model list of essential medicines was 0.5 US$) [3]. Thus, joint procurement of injectable medicines and single-use injection devices should not lead to a substantial increase of essential medicines expenditures in developing and transitional countries [3]. The national list of essential medicines must play a role in ensuring that the injectable medicines included are limited to the minimum so that unnecessary injections can be avoided. In this way, funds can be saved to finance syringes and needles for those injections that are necessary. Interventions to procure single-use injection devices to ensure injection safety are highly cost-effective health interventions [13].

While single-use injection devices should be made available in every health care facilities, they should also be made available in a way that ensures equity. In Burkina Faso, the poorest part of the population may not be able to assume the financial burden of single-use injection devices according to the Bamako Initiative. The use of cost recovery scheme could decrease service utilization by the general population and subsequently expose the poorest to unsafe injection practices and reuse of single-use injection devices [14]. The low frequency of reuse of injection devices in the 2000 injection safety survey suggests that the price of syringes and needles was not an obstacle to safe injections. However, the cost recovery issue is broader than the specific problem of access to injection devices. Equitable financing mechanisms avoiding user's fees for poor people should facilitate access to safe health care, essential medicines and injection devices for the poorest part of the population.

The generalized use of single-use injection devices in preventive and curative services has not led to major side effects in Burkina Faso. Excessive availability of injectable medicines can increase irrational use of injections and restricted access to injectable medications is associated with a reduction of injection overuse [15]. However, in Burkina Faso, the indicators selected to measure the use of injectable medicines did not suggest that the increased use of single-use injection devices led to an increase of the irrational use of injections. The implementation of the national essential medicines policy promoted the appropriate use of injectable medicines through limiting the number of injectable medicines on the national list of essential medicines and through training prescribers on the appropriate use of medicines. While an increased access to syringes and needles did not affect injection frequency, final disposal of used syringes remained a problem [7]. Making injections safe to the recipients should be the first priority from a public health point of view as the burden of disease associated with unsafe sharps waste disposal is of lower magnitude than the one associated with reuse of injection devices in absence of sterilization [2,16]. Nevertheless, a national sharps waste management policy that is planned, costed and budgeted is needed along with training of the health care workers to ensure appropriate sharps waste management.

Increased access to injection devices is not the only explanation that may account for the improvement of injection practices in Burkina Faso. Increased awareness regarding risks of transmission of pathogens, including HIV, through unsafe injections among the population may have played a role [7,17,18]. In 1994, a study reported that 16.9% of the pregnant women, 25.4% of the long-distance truck drivers and 11.5% of the sex-workers interviewed knew about the risk of HIV transmission through contaminated syringes and needles [17]. In 2000, a second study indicated that 60.4% of the persons randomly selected in Bobo-Dioulasso, the second largest city in Burkina, knew about the possibility of HIV transmission through injections [18]. While these two studies were carried out in different populations, an increase in the awareness regarding the risk of HIV transmission through injections cannot be excluded [7]. This awareness of risks may have increased consumers' demand for safe single-use syringes. In areas of the world where the awareness regarding unsafe injections is low (e.g., South Asia), [1] good geographical access to injection devices may not be associated with safe practices.

This programme review suffers from three main limitations. First, the 1995 and 2000 assessments used different methodologies and the 1995 assessment did not use standard WHO methods [7] However, three elements suggest that the apparent improvement between 1995–1996 and 2000 is real. The proportion of use of sterile devices observed in urban facilities in 1995 (80%) is still below the lower 95% confidence limit of the proportion of use of sterile devices reported nationwide in the 2000 assessment (85%). The majority of primary health care facilities in Burkina Faso are located in rural area where the proportion of use of sterile devices was 11% in 1995, contrasting with the 96% national average in 2000. The methodology used in 1995 was a convenience sample likely to have excluded remote primary health care facilities, which could have lead to an optimistic assessment of injection practices. Second, we used the availability of selected types of injection devices during the field visit as an indicator of access. This method may not have captured shortages that would have occurred before or after the visit. However, this indicator was chosen as (1) it is compatible with other access indicators used to monitor essential medicine policies [19] and (2) it was simple, reproducible and objective. Third, this assessment focused only on the public sector. Thus, we cannot rule out that the cost recovery scheme in place in public health care facilities would have driven patients to the private sector. However, this is unlikely to have happened because Burkina Faso does not have an major informal private sector [14] and because the formal private sector has user's fees that are generally higher than those in place in the public sector.

One element of the strategy to ensure injection safety is a continuous availability of sufficient quantities of injection devices in health-care facilities [20]. WHO, UNICEF and UNFPA reaffirmed the need to supply auto-disable syringes and safety boxes together for all consignments of vaccines [5]. Similarly, WHO developed a strategy to ensure that special attention is paid for the safe administration of all types of injections in health care services. WHO recommends that injection device security is ensured in all health care facilities, including therapeutic services, so that injectable medicines, diluents, single-use injection devices and safety boxes are supplied in timely manner in adequate quantities [6]. To practically assist procurement officers and pharmacists in procuring injection devices of good quality, WHO developed a procurement guide, [21] a quality assurance guide for injection devices [22] and a procedure for assessing, in principle, injection devices for the procurement by United Nations agencies [23]. Following this new policy through use of these tools should ensure injection device security. Injection device security, along with behaviour change and sharps waste management should prevent injection-associated infections in the future. Efforts to reduce overuse of injections should continue and be monitored using standardized indicators [24].

## Conclusion

In Burkina Faso, establishing pharmaceutical depots next to health care facilities through the national policy of essential medicines increased access to safe injection devices and contributed substantially to safer injection practices along with other factors, including an increased consumer demand for safe injection devices. The better access to single use injection devices was not parallelled by an increase in injection prescriptions. However, health care waste management policies need to address the increased amount of sharps waste generated.

## Competing interests

The author(s) declare that they have no competing interests.

## Authors' contributions

SL who was the principle investigator and writer of the article has shared with JT, the conception of the protocol and study design. PS participated in the formulation of the assessment tool, provided comments on the assessment tool and the writing of the study. YH wrote the initial terms of reference of the study, participated in the analysis of the data and in the writing of the paper. KH supervised all aspects of the investigations and of the writing.

## Pre-publication history

The pre-publication history for this paper can be accessed here:


